# Altered rumen bacterial flora is associated with increased lipogenesis of adipose tissue in obese dairy cows before calving

**DOI:** 10.1186/s40168-026-02343-7

**Published:** 2026-02-06

**Authors:** Chenxu Li, Guowen Liu, Yuting Yang, Zhaoxin Shi, Qi Shao, Zhiyuan Fang, Yuxiang Song, Wenwen Gao, Lin Lei, Xiliang Du, Xinwei Li

**Affiliations:** State Key Laboratory for Diagnosis and Treatment of Severe Zoonotic Infectious Diseases, Key Laboratory for Zoonosis Research of the Ministry of Education, Institute of Zoonosis, and, College of Veterinary Medicine , Jilin University, Changchun, 130062 China

**Keywords:** Dairy cows, Rumen bacterial flora, Prepartum obesity, Volatile fatty acids, Glucose and lipid metabolism

## Abstract

**Background:**

Prepartum obesity predisposes dairy cows to a higher risk of postpartum metabolic disorder. Volatile fatty acids (VFA) produced through ruminal microbial fermentation of feed substrates serve as a key form of energy for dairy cows. However, the precise mechanisms through which the rumen microbiota promote adipocyte lipid accumulation in obese dairy cows remain to be elucidated. Thus, the aim of this study was to investigate the mechanisms by which rumen microbiota regulates prepartum obesity in dairy cows.

**Results:**

Plasma glucose, insulin, triglyceride, and free fatty acids were greater in obese dairy cows. In the adipose tissue, the triglyceride content and expression of genes involved in lipid synthesis were higher in obese dairy cows. In the liver, the expression of genes involved in gluconeogenesis and lipid synthesis was higher in obese dairy cows. The ruminal total VFA, acetate, and propionate were higher in obese dairy cows compared to normal cows. The 16S rRNA gene analysis revealed that rumen bacteria, including *Tidjanibacter inops*_*A*, *Rikenella massiliensis*, *Papillibacter cinnamivorans*, and *Parabacteroides merdae*, were enriched in the rumen of obese dairy cows. Enrichment of these bacteria promoted carbohydrate degradation and VFA production. The metabolome analysis showed that obese dairy cows had elevated citric acid level in the rumen, which was positively associated with body condition score, body weight, adipocyte diameter, ruminal VFA concentration, and the abundance of VFA-producing bacteria.

**Conclusions:**

Our results suggest that rumen bacterial flora in prepartum obese dairy cows supply more VFA to the host, which may induce lipid deposition in adipocytes.

Video Abstract

**Supplementary Information:**

The online version contains supplementary material available at 10.1186/s40168-026-02343-7.

## Background

Maternal obesity is an increased risk factor for pregnancy-related disorders, including gestational hypertension, preeclampsia, and gestational diabetes [1, 2]. The transition period is crucial for dairy cow health, milk production, and farm profitability, because 75% of all infectious diseases and metabolic disorders emerge during this phase [3, 4]. Dairy cows with a high body condition score (BCS; BCS ≥ 4) in the prepartum experience a sharper reduction in postpartum dry matter intake (DMI), resulting in a severe negative energy balance, which makes them more susceptible toward metabolic diseases, such as fatty liver and ketosis [5, 6]. For instance, obese cows (BCS ≥ 4) at calving tend to have a risk of ketosis that is 1.6 times higher compared to normal cows (BCS = 3.25–3.75) [7]. Periparturient obesity in cows is more susceptible to hypocalcemia, hypomagnesemia, hypophosphatemia, and hypoglycemia [8]. Moreover, obesity in dairy cows is detrimental to birth outcomes, including low birth weight and increased offspring inflammation [9].

Volatile fatty acids (VFA) produced in the rumen are the primary energy precursors in ruminants [10]. Acetate, propionate, and butyrate account for 95% of the total VFA, and acetate and butyrate are substrates as precursors of lipids [11, 12]. During positive energy balance, acetate is essential for de novo lipogenesis in adipose tissue because it serves as a 2-carbon donor for synthesis of malonyl-CoA [13, 14]. Propionate is the only glucogenic VFA in dairy cows and contributes as much as 60 to 74% of the carbon for gluconeogenesis [15]. Of note, dairy cows with a high BCS in the prepartum demonstrate elevated blood glucose level [16, 17]. Because acetate, butyrate, and glucose are used to de novo lipogenesis in adipose tissue, it is possible that the increased lipid deposition observed in adipose tissue of obese cows is due to elevated ruminal VFA levels.

In ruminants, VFA contribute more than 70% of the metabolic energy requirements of the host, and approximately 90% of the amino acids available in the small intestine are derived from ruminal microbial protein [18, 19]. It has been reported that there are significant changes in the rumen bacteria composition in dairy cows over parturition and early lactation [20]. Dairy cows with higher BCS have a greater abundance of rumen bacterial genera related to cellulose decomposition and VFA production, such as *Veillonellaceae*_*UCG*_*001*, *Prevotellaceae*_*YAB2003*_*group*, and *Ruminococcus*_*gauvreauii*_*group* [21]*.* Additionally, supplementing early-lactating cows with *Prevotella bryantii* 25A increased ruminal ammonia-N (NH_3_-N) and VFA levels and milk fat concentration [22]. More importantly, a body of evidence shows that rumen and gut microbiota helps to harvest energy and increase host fat storage [23–25]. Although obesity is prevalent in dairy cows before calving [26], the mechanisms by which rumen microbiota affect fat deposition in adipose tissue remain poorly understood. In the present study, we hypothesized that alterations in the composition and function of rumrn microbiota contribute to increased lipid storage in adipose tissue of dairy cows during the prepartum period. Thus, our objectives were to assess the associations between rumen microbiota and lipogenesis in adipocytes to better understand how rumen microbiota contributes to lipid storage in adipose tissue commonly observed in dairy cows before calving.

## Methods

### Animals

**Fig. 1:**
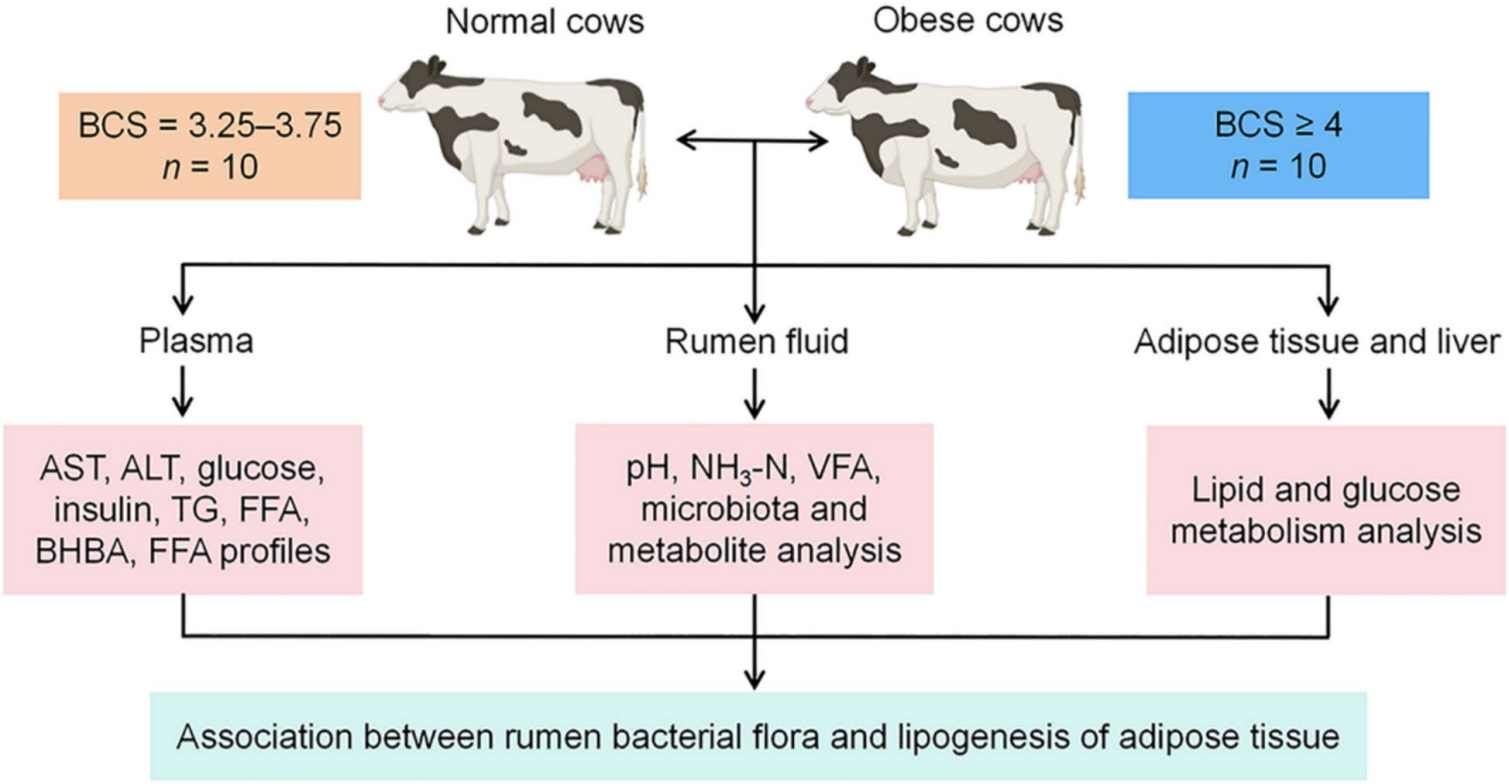
Experimental design flow chart. Twenty cows were allocated to either the normal (BCS = 3.25–3.75; *n* = 10) or obese groups (BCS ≥ 4; *n* = 10) according to their BCS at 14 days before calving. Samples of plasma, rumen fluid, adipose tissue, and liver were collected to assess blood metabolic parameters, rumen microbiota and metabolites, rumen fermentation parameters, as well as lipid and glucose metabolism in adipose tissue and liver. *BCS* body condition score, *AST* aspartate aminotransferase, *ALT* alanine aminotransferase, *TG* triglyceride, *FFA* free fatty acids, *BHBA* beta-hydroxybutyrate, *NH*_3_-*N* ammonia-N, *VFA* volatile fatty acids

The protocol for the current study was approved by the Jilin University Ethics Committee on the Use and Care of Animals (Changchun, China, SY202303301). Based on the expected calving date, twenty clinically healthy multiparous Chinese Holstein cows (median parity = 3, range = 2–4) were selected and allocated to either the normal (BCS = 3.25–3.75; *n* = 10) or obese groups (BCS ≥ 4; *n* = 10) according to their BCS as previously described [5]. The average (mean ± SD) BW at − 14 days relative to parturition was 727 ± 18 kg and 812 ± 21 kg in normal and obese cows, respectively. Dry cows were housed in a free stall barn. Dry matter intake for each group was calculated as the difference between daily feed offered and feed refused for 3 days before sample collection. Cows were fed a total mixed ration (TMR) diet (Table S1) during the prepartum period, with free access to water during the entire study. The experimental design is shown in Fig. 1.

### Sample collection

All samples were collected at − 14 days relative to parturition. Before the morning feeding, blood from the coccygeal vein was collected into evacuated tubes containing sodium heparin (YL063, Jiangsu Yuli Medical Instrument Co., Ltd., Taizhou, China). Blood samples were centrifuged at 3500 × *g* for 15 min at 4 °C and plasma was harvested and stored at − 80 °C until further analysis.

Rumen fluid samples were collected from each cow using a flexible esophageal tube (A1141K; Anscitech Co., Ltd., Wuhan, China) 3 h after the morning feeding. After discarding the first 200 mL of fluid to minimize saliva contamination, approximately 50 mL of rumen fluid was collected. Four layers of sterile gauze were used to filter the rumen fluid samples and the ruminal pH was determined immediately with a portable pH meter (PH200, Ruizhen Electronic Technology Co., Ltd., Shanghai, China). After pH measurement, two 5 mL rumen fluid samples were immediately frozen in liquid nitrogen and stored at − 80 °C until DNA extraction and metabolite detection. Two 5-mL aliquots were prepared: one mixed with 100 µL of 50% sulfuric acid for NH_3_-N determination, and the other with 1 mL of 25% metaphosphoric acid for VFA analysis. Both were stored at − 20 °C prior to measurement.

Before liver biopsy, the intercostal space was shaved, sanitized with iodine scrub and 75% alcohol, and anesthetized with subcutaneous injection of 2% lidocaine HCl (1,366,013; Sigma-Aldrich Co., St. Louis, MO, USA). The biopsy needle (BN-ORC-2/GT20; GMT medical, Beijing, China) was then inserted through the intercostal muscle layers and into the liver to obtain tissue samples. Adipose tissue biopsy from the tail-head region was collected from each cow by blunt dissection through an incision in the skin and subcutaneous layers, followed by washing with saline solution. The adipose tissue and liver samples from each cow were all divided into two portions: one portion was frozen in liquid nitrogen and stored at − 80 °C, and the other portion was fixed with 4% paraformaldehyde (P1110; Solarbio, Beijing, China). Tissue samples stored at − 80 °C were placed on dry ice, rapidly transported to the laboratory, and subsequently divided into three portions: one portion was placed in TriQuick Reagent (R1100; Solarbio) to maintain RNA stability and then stored at − 80 °C for RNA extraction, and the remaining two portions were directly stored at − 80 °C until triglyceride (TG) determination and protein extraction.

### Blood parameters

According to the manufacturer’s instructions, plasma concentrations of glucose, insulin, TG, free fatty acids (FFA), as well as the activities of aspartate aminotransferase (AST) and alanine aminotransferase (ALT) were measured with a microplate Reader (Multiskan 51,119,000; Thermo Fisher Scientific, Waltham, MA, USA) using commercially available kits (glucose, cat. no. A154-1–1; insulin, cat. no. H203-1–2; TG, cat. no. A110-1–1; FFA, cat. no. A042-2–1; AST, cat. no. C010-2–1; ALT, cat. no. C009-2–1; Nanjing Jiancheng Bioengineering Institute, Nanjing, China). The plasma beta-hydroxybutyrate (BHBA) was measured using a Hitachi 3110 autoanalyzer (Hitachi, Tokyo, Japan) with a commercially available kit (RB1008 Randox Laboratories, Crumlin, UK).

### Blood FFA profiles

Plasma total lipids were extracted and fatty acid methyl esters (FAMEs) were prepared as previously described [27]. The analysis of FAMEs was conducted using a gas chromatography (Agilent 8890; Agilent Technologies, Santa Clara, CA, USA), and nitrogen gas was employed as a carrier at a consistent flow rate of 1.1 mL/min. The chromatographic reference conditions were as follows: fused silica capillary column (100 m × 0.25 mm, 0.20 µm) was used; the initial column temperature was set at 150 °C and held at this temperature for 5 min, then increased to 200 °C at a rate of 2 °C/min for 10 min, with a subsequent increase to 220 °C at a rate of 5 °C/min and kept at this temperature for 5 min; the injector and detector temperatures were 260 °C and 280 °C, respectively. The measurement of the peak area was conducted by Open Lab CDS (Agilent Technologies), and the identification of the peaks was accomplished through the comparison of their retention times with FAME standards (18,919-1AMP; Sigma-Aldrich Co.).

### Rumen fermentation parameters

Rumen fluid samples preserved with sulfuric acid and metaphosphoric acid were thawed and then transferred into 2 mL tubes. Then, the collected samples were centrifuged at 30,000 × *g* for 20 min at 4 °C, and the supernatant from samples in sulfuric acid was used to analyzed NH_3_-N using a colorimetric assay described by Chaney and Marbach [28]. The supernatant of rumen fluid in metaphosphoric acid was analyzed for VFA using a gas chromatograph (GC-14B; Shimadzu, Kyoto, Japan; capillary column film thickness: 30m × 0.32 mm, 0.25 µm; column temperature 130 °C; injector temperature 180 °C; detector temperature 180 °C) [29].

### TG measurement

Approximately 20 mg of adipose tissue and liver samples were homogenized by radioimmunoprecipitation assay lysis buffer (C1053; Applygen Technologies Inc., Beijing, China) to determine total protein concentration using the bicinchoninic acid (BCA) assay (P1511; Applygen Technologies Inc.). A separate portion collected supernatant was heated in a water bath at 70 °C for 10 min, and was centrifuged at 2000 × *g* for 5 min at 4 °C. The TG concentration in the supernatant was determined using an enzymatic kit (E1013; Applygen Technologies Inc.).

#### Histological staining

Samples of adipose tissue and liver fixed with 4% paraformaldehyde were embedded in paraffin and cut into sections with a thickness of 4 µm. Tissue sections were dewaxed with xylene, followed by rehydration through a series of decreasing alcohol concentrations, and were subsequently stained with hematoxylin and eosin (H&E). In the Oil-Red O staining experiment, liver samples were frozen in optimal cutting temperature compound (OCT; Sakura Finetek, Torrance, CA), sectioned into 8 μm slices at − 18 °C, and fixed with 75% alcohol at room temperature for 15 min. Then, slides were stained with Oil-Red O (0625; Sigma-Aldrich Co., St Louis, MO, USA) and counterstained with hematoxylin.

### Real-time quantitative PCR

Total RNA was extracted from adipose tissue and liver using TriQuick Reagent (R1100; Solarbio). The RNA concentration and quality were evaluated using a Nanophotometer N50 Touch (Implen GmbH, Munich, Germany), and the RNA integrity was analyzed by gel electrophoresis (1% agarose gel). Then, the RNA was reverse transcribed into cDNA using the reverse transcription kit (RR047A; TaKaRa, Tokyo, Japan). The mRNA expression was measured using the SYBR Green QuantiTect RT-PCR Kit (RR420A; Takara) via a 7500 RT-PCR system (Applied Biosystems Inc., Waltham, MA, USA). The normalization of the relative expression levels of each target gene was conducted using two reference genes, actin beta (*ACTB*) and *GAPDH*, and calculated through the 2^−∆∆CT^ method. The primer sequences for each target gene and the reference gene are shown in Table S2. The *GAPDH* and *ACTB* were assessed for their stability and were not affected by the different experimental conditions, which were validated as stable control genes.

### Western blotting

Total protein was extracted from adipose tissue using RIPA lysis buffer (P0013B; Beyotime, Shanghai, China). Protein content was quantified using the BCA method according to the manufacturer’s instructions (P1511; Applygen Technologies). Subsequently, the protein was diluted with 5 × loading buffer for denaturation at 95 °C for 8 min, separated by sodium dodecyl sulfate-PAGE (8% stacking gel and 10% separating gel), and transferred to polyvinylidene fluoride (PVDF) membrane (0.45 µm). After being blocked with 3% BSA for 4 h at room temperature, membranes were incubated overnight at 4 °C with specific antibodies against protein sterol regulatory element-binding protein 1C (SREBP-1C; 1:1,000; NB100–2215; Novus Biologicals, Littleton, CO, USA), hormone-sensitive lipase (HSL; 1:1,000; AF6403; Affinity Biosciences Ltd., Jiangsu, China), phosphorylated-HSL (p-HSL; 1:1,000; AF2350; Affinity Biosciences Ltd.), adipose triglyceride lipase (ATGL; 1:1,000; Ab99532; Abcam, Cambridge, MA, USA), protein kinase B (AKT; 1:1,000; cat. no. 9272; Cell Signaling Technology Inc., MA, USA), p-AKT (1:1,000; cat. no. 4060; Cell Signaling Technology Inc.), and ACTB (1:2,000; Ab8226; Abcam), respectively. Subsequently, the PVDF membranes were incubated with horseradish peroxidase conjugated goat ant-rabbit (1:5,000; ab205718, Abcam) or mouse immunoglobulin at room temperature for 45 min and the protein bands were detected using a Tanon imaging system (Tanon 4600, Tianneng Co., Ltd., Shanghai, China) with an enhanced chemiluminescence solution (WBULS0500, Millipore, Bedford, MA, USA). All bands were analyzed using Image-Pro Plus 6.0 (Media Cybernetics, Rockville, MD, USA) and target proteins normalized to ACTB.

### Rumen microbial DNA extraction, PCR amplification, and 16S rRNA third-generation full-length sequencing

Microbial DNA was extracted from rumen fluid samples using the cetyl-trimethyl ammonium bromide (CC3991; Coolaber, Beijing, China) plus bead-beating method [30]. The finally obtained DNA concentration and purity were determined by a NanoDrop 1000 spectrophotometer (Nyxor Biotech, Paris, France), and DNA integrity was checked on 1% agarose gel. The V1–V9 region of the bacteria 16S ribosomal RNA gene were amplified using an ABI GeneAmp® 9700 PCR thermocycler (ABI, CA, USA) by primer pairs 27F (5′-AGRGTTYGATYMTGGCTCAG-3′) and 1492R (5′-RGYTACCTTGTTACGACTT-3′). The PCR reactions were conducted using the following program: 95 °C for 2 min, followed by 27 cycles at 95 °C for 30 s, 55 °C for 30 s, and 72 °C for 60 s, and a final extension at 72 °C for 5 min, and end at 4 °C. PCR reactions were performed in triplicate in the 20 µL mixture and amplicons were extracted from 2% agarose gels and purified using the DNA gel extraction kit (AP-GX-250; Axygen Biosciences, Union City, CA, USA) following the manufacturer’s instructions and quantified using the Quantus™ Fluorometer (E6150; Promega, Madison, USA). Purified amplicons were pooled in equimolar and sequenced on an PacBio Sequel II platform (Pacific Biosciences, Menlo Park, CA, USA) according to the standard protocols by Shanghai Biozeron Biotechnology Co., Ltd. (Shanghai, China). OTUs with the 98.65% similarity level were clustered using UPARSE (http://drive5.com/uparse/) and chimeric sequences were removed using UCHIME (http://drive5.com/uchime). Low abundance OTUs were filtered, and normalized OTUs were generated. The taxonomy of each OTU representative sequence was analyzed by Ribosomal Database Project (RDP) Classifier (http://rdp.cme.msu.edu/) against the SILVA (SSU132) 16S rRNA database using a confidence threshold of 70%. For rumen microbial data, α diversity was calculated including Simpson and Shannon with Mothur (v1.30.1). Beta diversity was determined to compare the similarity and dissimilarities between normal and obese groups by principal coordinate analysis (PCoA) based on Bray–Curtis dissimilarity. We calculated differences in β diversity using PERMANOVA (adonis) tests using the “vegan” package with 999 permutations in R (v.3.5.1). Rumen bacterial phyla and genera were compared using Wilcoxon rank-sum test with the Benjamini-Hochberg (BH) adjusted *P* value < 0.05 being considered as significantly different. Linear discriminant analysis (LDA) effect size (LEfSe) analysis was used to compare rumen bacterial species, and significant differences identified by a *P* < 0.05 and an LDA score > 2.

### Shotgun sequencing

The metagenome libraries were constructed by the TruSeq Nano DNA Library Preparation Kit (FC-121–2001; Illumina, San Diego, CA, USA). The metagenome libraries were sequenced on an Illumina NovaSeq6000 platform with paired-end 150 bp (PE150) mode. Low quality reads and bovine genome (bosTau8 3.7, 10.18129/B9.bioc.BSgenome.Btaurus. UCSC.bosTau8) were removed using Trimmomatic (https://github.com/topics/trimmomatic) and BWA (http://bio-bwa.sourceforge.net/). Then, MEGAHIT (https://github.com/voutcn/megahit) was used to assemble the obtained clean reads. We used Prodigal (http://prodigal.ornl.gov/) to predict the open reading frames (ORFs) of assembled contigs and took advantage of CD-HIT (http://www.bioinformatics.org/cd-hit/) to cluster contigs from each sample’s assembly based on 95% cutoff sequencing identity. The longest sequence in each cluster was used as a representative sequence to construct a non-redundant gene set. Salmon software (https://combine-lab.github.io/salmon/) was used to get the nunber of reads number each gene. The abundance of each gene was normalized to transcripts per million (TPM). Gene functional annotations were obtained by aligning to NR (https://www.ncbi.nlm.nih.gov/), eggNOG (http://eggnog5.embl.de/), KEGG (http://www.genome.jp/kegg/), and CAZy databases (http://www.cazy.org/) using Diamond (https://github.com/bbuchfink/diamond). Microbial functions at level 2 of the KEGG pathways, KEGG ortholog (KO) genes, CAZymes, and eggNOG functional categories were subjected to comparison between two groups using the Wilcoxon rank-sum test with the BH adjusted *P* value < 0.05 considered significantly different. The LEfSe was performed to identify the characteristic level 3 KEGG pathways.

### Untargeted metabolomics

A 100 μL rumen liquid sample was mixed with a 400 μL methanol:water (4:1, v/v) solution to extract the metabolites. The mixtures were vortexed for 30 s, followed by extraction in an ultrasonic bath (KQ5200DE; Kunshan Ultrasonic Instrument Co., Ltd., China) at a temperature of 5 °C with an ultrasonic frequency of 40 kHz for 30 min, and subsequent incubation at − 20 °C for 30 min. The resulting solution was then subjected to centrifugation at 13,000 × *g* for 15 min at 4 °C to isolate the protein precipitate, after which the supernatants were transferred and dried using a vacuum evaporator. The resulting concentrated product was resuspended in 100 µL of water:acetonitrile (1:1, v/v). The liquid chromatograph mass spectrometer (LC–MS) analysis was performed using an ultra-high-performance liquid chromatography (UHPLC) system (1290, Agilent Technologies, Waldbronn, Germany) with a UPLC HSS T3 column (100 mm × 2.1 mm, 1.8 μm) coupled to Q Exactive mass spectrometer (Orbitrap MS, Thermo Fisher Scientific, San Jose, CA, USA). The column temperature was set at 40 °C, with an injection volume of 2.0 µL. Eluent A was prepared by combining water and acetonitrile in a 5:95 ratio, while eluent B was prepared by mixing acetonitrile, 2-propanol, and water in a 47.5:47.5:5 ratio. Mass spectrometric data were collected in both positive and negative modes using an electrospray ionization source. To assess the consistency of the system, quality control (QC) samples were prepared by combining all rumen fluid extraction aliquots and injected at regular intervals throughout the analytical run. The raw data underwent processing using Progenesis QI software (Waters, Milford, USA) to perform baseline filtering, peak identification, integration, retention time (RT) correction, peak alignment, resulting in a data matrix comprising RT, m/z, and peak intensity. To mitigate errors arising from sample preparation and instrument instability, the response intensity of the sample mass spectrum peak was normalized using the sum normalization method. Additionally, variables in QC samples exhibiting a relative standard deviation (RSD) exceeding 30% were excluded from the final data matrix, which was subsequently used for further analysis. The principal component analysis (PCA) and partial least squares discriminant analysis (PLS-DA) were performed with SIMCA-P software (v.13.0, Umetrics, Umea, Sweden). The Wilcoxon rank-sum test was performed between the two groups, differential metabolites were selected based on a BH adjusted* P* value < 0.05 and the variable importance in the projection (VIP) > 1. Volcano plots were used to filter metabolites of interest based on log2 (fold change; FC) and − log10 (*P* value) of metabolites with “ggplot2” in R. Metabolite source analysis was conducted using the MetOrigin platform (http://metorigin.met-bioinformatics.cn/). To identify metabolic pathways, enrichment analysis in MetOrigin was applied to metabolites from each cluster.

### Correlation analysis

All correlation analyses were performed using Spearman’s rank correlation method, with a BH adjusted *P* value < 0.05 and |rho|> 0.60 considered statistically significant. The correlation network was visualized via Cytoscape (http://www.cytoscape.org). Additionally, the correlation heat map was generated using the “pheatmap” package within the R.

### Statistical analysis

Data analysis was carried out using SPSS 22.0 software (SPSS Inc., Chicago, USA) or GraphPad Prism 10.0 (GraphPad Software Inc., San Diego, USA). The sample distribution was determined by the Shapiro–Wilk and Levene tests, respectively. The independent sample *t* test was used to evaluate statistical significance for normal distribution. For the nonparametric tests, the Wilcoxon rank-sum test was used to evaluate statistical significance. Data were expressed as means ± standard deviation (SD), *P* value < 0.05 was considered statistically significant, and *P* value < 0.01 was considered extremely significant.

## Results

### Metabolic parameters

Compared to normal cows, BCS and BW were higher in obese cows (Fig. S1A and B; *P* < 0.01), whereas DMI was similar (Fig. S1C; *P* > 0.05). Plasma biomarkers of liver function including ALT and AST had no difference between the two groups (Fig. S1D and E; *P* > 0.05). Plasma glucose, insulin, TG, and FFA were greater in obese cows (Fig. S1F–I; *P* < 0.01), whereas BHBA did not differ (Fig. S1J; *P* > 0.05).

### Lipid and glucose metabolism of adipose tissue and liver and blood FFA profiles

**Fig. 2:**
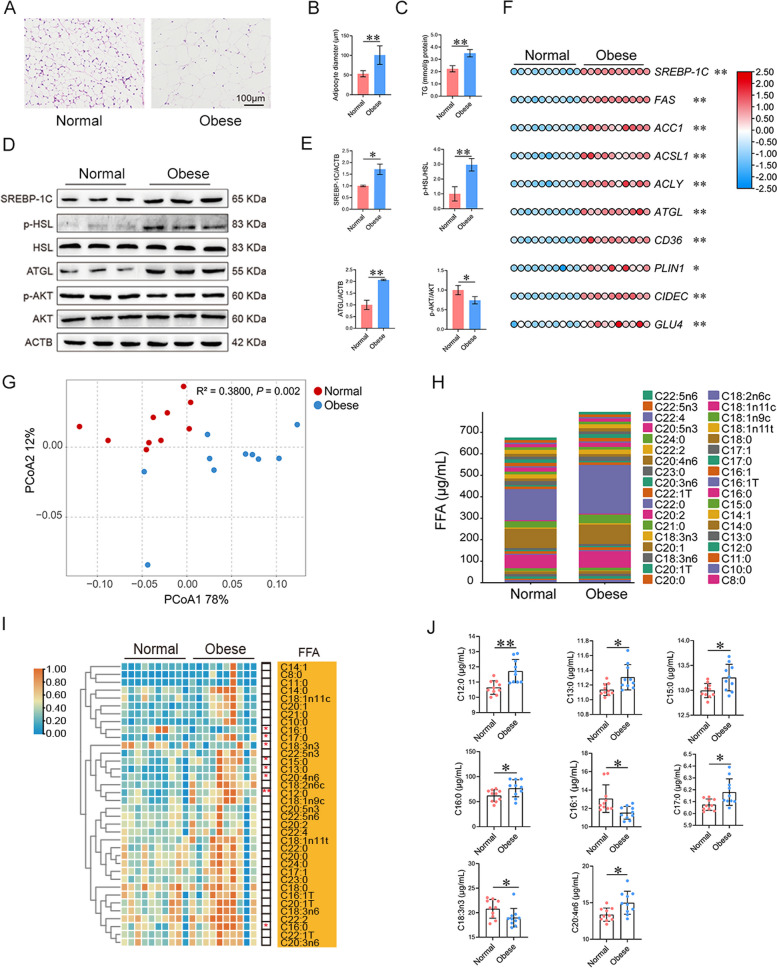
Differential lipid and glucose metabolism of adipose tissue and blood FFA profiles between normal and obese dairy cows. **A** Representative images of hematoxylin–eosin (H&E) staining of the adipose tissue. **B**, **C** Adipocyte diameter and TG content in the adipose tissue. **D** Representative blots of SREBP-1C, p-HSL, HSL, ATGL, p-AKT, AKT, and ACTB. **E** Quantification of protein levels of SREBP-1C, p-HSL, ATGL, and p-AKT. **F** Heatmap of the abundance of genes related to lipid and glucose metabolism in the adipose tissue. **G** The PCoA analysis based on plasma FFA profiles. **H** Average concentrations of plasma FFA. **I** Heatmap of the relative concentrations of plasma FFA profiles. **J** Significantly different FFA in plasma. *n* = 10 per group. Significant differences were tested using the independent samples *t* test. Data with error bars were expressed as mean ± SD. *P < 0.05, **P < 0.01

The adipocyte diameter (AD) and TG content were higher in the adipose tissue of obese cows compared to normal cows (Fig. 2A–C; *P* < 0.01). Compared to normal cows, the protein abundance of SREBP-1 and ATGL, and the ratio of p-HSL to HSL in the adipose tissue were higher in obese cows (Fig. 2D and E; *P* < 0.05 or* P* < 0.01). In contrast, the ratio of p-AKT to AKT was lower in obese cows (Fig. 2D and E; *P* < 0.01). In the adipose tissue, the mRNA abundance of genes involved in lipid synthesis (*SREBP-1C*, *FAS*, *ACC1*, *ACSL1*, *ACLY*), lipid hydrolysis (*ATGL*), lipid droplet formation (*PLIN1* and *CIDEC*), as well as fatty acid and glucose transportation (*CD36* and *GLU4*) was greater in dairy cows with obesity (Fig. 2F; *P* < 0.05 or *P* < 0.01). In the liver, the H&E staining revealed that obese cows displayed increased lipid accumulation (Fig. S2A). Furthermore, Oil-Red O staining showed that more lipid droplets were observed in the liver of dairy cows with obesity (Fig. S2A). The hepatic TG content of obese cows was much higher than that of normal cows (Fig. S2B; *P* < 0.01). The hepatic mRNA abundance of genes regulating lipid synthesis (*SREBP-1C*, *ACSL1*, *ACSS2*, *ACLY*, *FAS*, and *ACC1*) and gluconeogenesis (*G6P1*, *PCK1*, and *PC*) was higher in obese cows than in normal cows (Fig. S2C; *P* < 0.01 or *P* < 0.05). In contrast, the abundance of genes involved in lipid oxidation (*CPT1* and *ACOX1*) was lower in obese cows (*P* < 0.01). PCoA analysis revealed a significant difference in plasma FFA profiles between normal and obese cows (Fig. 2G; *P* < 0.01). A total of 36 FFA were detected in plasma samples (Fig. 2H and Table S3). The C12:0, C13:0, C15:0, C16:0, C17:0, and C20:4n6 were higher, whereas C16:1 and C18:3n3 were lower in obese cows compared to normal cows (Fig. 2I and J;* P* < 0.05). These data indicate that dairy cows with obesity display lipid accumulation in adipose tissue and liver and increased FFA in the blood.

### Rumen fermentation parameters and bacterial composition

**Fig. 3:**
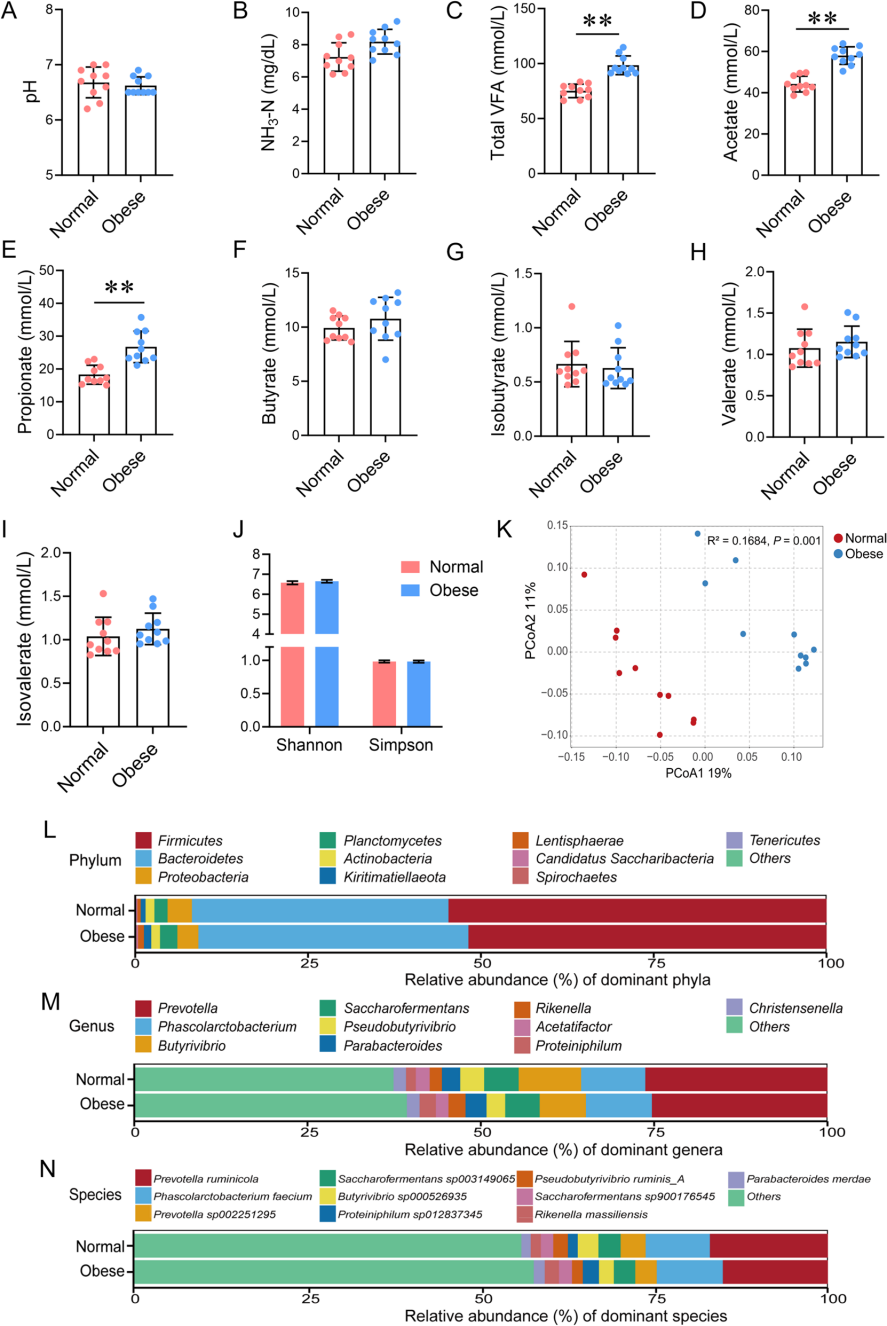
Differential rumen fermentation parameters and bacterial composition between normal and obese dairy cows. **A** Ruminal pH. **B** Concentration of ammonia-N (NH_3_-N) in the rumen fluid. **C**–**I** Concentrations of total VFA, acetate, propionate, butyrate, isobutyrate, valerate, and isovalerate. **J** Shannon and Simpson indexes of ruminal bacterial communities. **K** PCoA of rumen bacterial communities. **L**–**N** Community biplot analysis at the phylum, genus and species levels. *n* = 10 per group. Significant differences were tested using the independent samples *t* test. Data with error bars were expressed as mean ± SD. *P < 0.05, **P < 0.01

The rumen fluid pH and the concentration of NH_3_-N between the two groups of dairy cows were not significantly different (Fig. 3A and B;* P* > 0.05). The total VFA, acetate, and propionate were higher in obese cows compared to normal cows (Fig. 3C–E;* P* < 0.01), whereas butyrate, isobutyrate, isovalerate, and valerate remained unchanged (Fig. 3F–I; *P* > 0.05). Feed fermentation in the rumen is mainly determined by bacteria. Hence, 16S rRNA third generation full length sequencing was used to evaluate rumen bacterial community composition. No significant differences were found in the Shannon and Simpson indices of the rumen bacterial flora between the two groups (Fig. 3J;* P* > 0.05). PCoA analysis showed distinct discrimination of bacterial composition between normal and obese cows (Fig. 3K;* P* < 0.01). Although overall bacterial taxa composition was similar between the two groups of dairy cows, notable differences were observed for the relative abundance of certain bacteria at the phylum, genus, and species levels (Fig. 3L–N). These data demonstrate that the alteration of rumen bacterial flora increases VFA production in dairy cows with obesity.

### Distinct rumen bacterial taxa and CAZymes genes between obese and normal cows

**Fig. 4:**
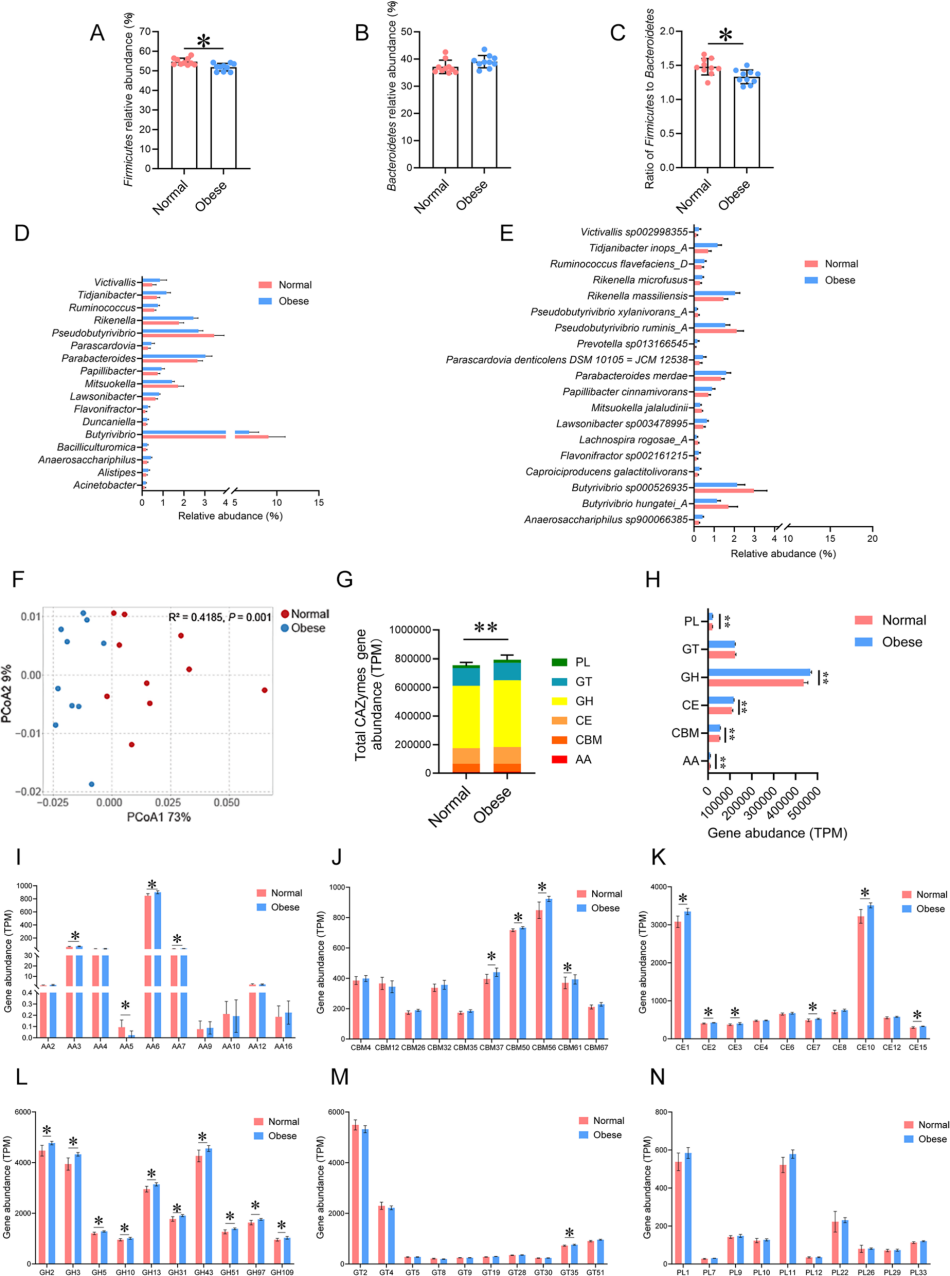
Differential bacterial taxa and CAZymes genes between normal and obese dairy cows. **A**–**C** Analysis of differences in *Firmicutes*, *Bacteroidetes* and the ratio *Firmicutes* to *Bacteroidetes*. **D** Abundance of significantly different bacterial genera. **E** Abundance of significantly different bacterial species. **F** The PCoA analysis based on all CAZyme genes. **G** Relative abundance of a total of CAZymes genes. **H** Analysis of differences in the relative abundance of the CAZymes genes at the class level. **I**–**N** Gene abundance of the top ten AA, CBM, CE, GH, GT, and PL family enzymes, respectively. *AA* auxiliary activity, *CBM* carbohydrate-binding module, *CE* carbohydrate esterase, *GH* glycoside hydrolase, *GT* glycosyltransferase, *PL* polysaccharide lyase. *n* = 10 per group. Significant differences were tested by linear discriminant analysis (LDA) effect size (LEfse) analysis (*P* < 0.05 and LDA > 2) for **E**. Significant differences were tested by the Wilcoxon rank-sum test for (**A**–**D**) and (**G**–**N**). Data with error bars were expressed as mean ± SD. *P < 0.05 and.**P < 0.01

The *Firmicutes* and the ratio of *Firmicutes* to *Bacteroidetes* in rumen of dairy cows with obesity were significantly lower than those of normal cows (Fig. 4A and C; *P* < 0.05), whereas *Bacteroidetes* did not differ (Fig. 4B; *P* > 0.05). At the genus level, the abundance of 14 bacterial genera was significantly higher (Fig. 4D; *P* < 0.05), whereas the abundance of *Pseudobutyrivibrio*, *Mitsuokella*, and *Butyrivibrio* was lower in obese cows compared to normal cows (Fig. 4D; *P* < 0.05). At the species level, 13 bacterial species showed significantly higher abundance in obese cows compared to normal cows (Fig. 4E; *P* < 0.05 and LDA > 2). Conversely, the abundance of two *Butyrivibrio* sp., two *Pseudobutyrivibrio* sp., one *Lachnospira* sp., and one *Mitsuokella* sp. was lower in obese cows relative to normal cows (Fig. 4E; *P* < 0.05 and LDA > 2). Metagenomic analysis was conducted to assess the functional and metabolic potential of the rumen microbial communities. For CAZyme profiles, a total of 335 genes encoding CAZymes were identified, including 12 auxiliary activities (AAs), 74 carbohydrate-binding modules (CBMs), 16 carbohydrate esterases (CEs), 132 glycoside hydrolases (GHs), 73 glycosyltransferases (GTs), and 28 polysaccharide lyases (PLs) (Table S4). PCoA of all CAZymes genes showed that normal and obese cows had different capacities to interact with carbohydrates (Fig. 4F; *P* < 0.01). The obese cows had a higher abundance of total CAZymes (Fig. 4G; *P* < 0.01), including AAs (Fig. 4H; *P* < 0.01), CBMs (*P* < 0.01), CEs (*P <* 0.01), GHs (*P* < 0.01), GTs (*P* > 0.05) and PLs (*P* < 0.01). Among the genes encoding CAZymes involved in deconstructing carbohydrates (Fig. 4I, K, L, and N), 1 was enriched in normal cows (1 AA; *P* < 0.05), whereas 19 were enriched in obese cows (10 GHs, 6 CEs, and 3 AAs; *P* < 0.05). Among the GTs (involved in carbohydrate synthesis; Fig. 4M), 1 was enriched in obese cows (*P* < 0.05), whereas no GTs were enriched in normal cows (*P* > 0.05). Regarding the CBMs (Fig. 4J), the noncatalytic CAZymes implicated in the breakdown of complex carbohydrates, no CBMs were found to be enriched in normal cows (*P* > 0.05), whereas 4 CBMs were enriched in obese cows (*P* < 0.05). Together, these results suggest that changes in rumen microbiota of dairy cows with obesity contribute to an enhanced ability for carbohydrate metabolism.

### Rumen microbial function

**Fig. 5:**
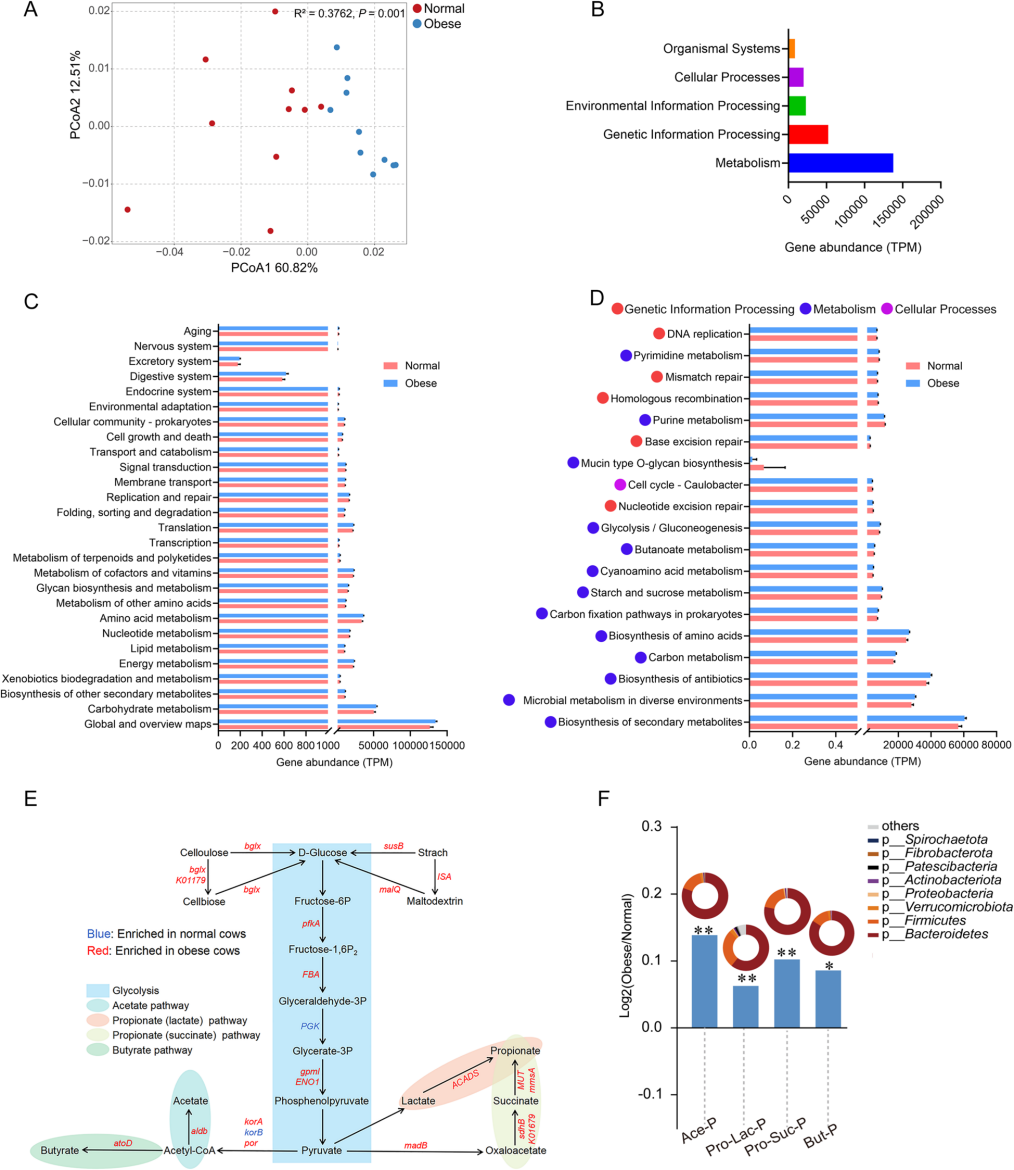
Comparison of rumen microbial functions between normal and obese dairy cows. **A** The PCoA analysis based on all KO genes. **B** Statistics of the first-level KEGG pathways. **C** Significantly different second-level KEGG pathways. **D** Significantly different third-level KEGG pathways. **E **Comparison of the relative abundance of KO enzymes enriched in the rumen microbiota regarding carbohydrate metabolism to produce VFA between the normal and obese groups. **F** Acetate, butyrate and propionate pathways of KEGG enzymes expressed as ratios of alignments of obesity versus normal (log2 ratio); and pie charts show the phylogenetic distribution of the pathways enriched in each group at phylum. The red font indicated enzyme genes enriched in obese cows while blue font indicated enzyme genes enriched in normal cows. *Ace*-*P* acetate production pathway, *Pro*-*Lac*-*P* propionate (lactate) pathway, *Pro*-*Suc*-*P* propionate (succinate) pathway, *But*-*P* butyrate production pathway. *n* = 10 per group. Significant differences were tested by the linear discriminant analysis (LDA) effect size (LEfse) analysis (*P* < 0.05 and LDA > 2) for (**D**). Significant differences were tested by the Wilcoxon rank-sum test for (**C**) and (**E**, **F**). Data with error bars were expressed as mean ± SD. *P < 0.05, **P < 0.01

PCoA of all KO genes showed that the rumen microbial functions were different between the two groups (Fig. 5A;* P* < 0.01). KEGG pathway analysis showed that a total of 262 endogenous third-level pathways were identified and classified as rumen microbial metabolic pathways (Table S5). These pathways were assigned to 5 first-level categories (Fig. 5B), including “Metabolism,” “Genetic Information Processing,” “Environmental Information Processing,” “Cellular Processes,” and “Organismal Systems.” When the secondary KEGG pathways were compared, a total of 27 pathways showed differences (Fig. 5C; *P* < 0.05). The category “Carbohydrate metabolism” tended to be higher in obese cows than in normal cows (Fig. 5C; *P* < 0.01). At the third-level pathways, a total of nine pathways, including one “Cellular processes” pathway, five “Genetic information processing” pathways and three “Metabolism” pathways, were significantly enriched in normal cows, whereas a total of ten “Metabolism” pathways were significantly enriched in obese cows (Fig. 5D;* P* < 0.05 and LDA > 2). Within the category “Carbohydrate metabolism,” “ko00010: Glycolysis/gluconeogenesis,” “ko00650: Butanoate metabolism,” and “ko00500: Starch and sucrose metabolism” were significantly enriched in obese cows (Fig. 5D; *P* < 0.05 and LDA > 2). Moreover, we used the eggNOG database to evaluate the functional differences between the two groups (Fig. S3). Twenty-three functional modules were obtained from these cows (Fig. S3A). The PCoA analysis of functional components showed a clear separation of normal and obese cows (Fig. S3B;* P* < 0.01). The category “Carbohydrate transport and metabolism” was also significantly enriched in obese cows (Fig. S3C). Subsequently, we conducted an analysis of genes responsible for encoding enzymes associated with the metabolic pathways of VFA production. We found that most of the genes involved in starch and cellulose degradation were enriched in obese cows (Fig. 5E; *P* < 0.05). Furthermore, the obese cows enriched for the pathways of acetate production (*aldb* gene), propionate production via succinate as intermediate (*K01679*, *sdhb*, *mmsA*, and *MUT* genes), propionate production via lactate as intermediate (*ACADS* gene), and butyrate production (*atoD* gene) (Fig. 5E; *P* < 0.05). *Bacteroidetes*, *Firmicutes*, and *Verrucomicrobiota* were the major phylum assigned to fermentation pathways producing these VFA (Fig. 5F). These results indicate that a higher potential capability to produce VFA exists in rumen microbiota of obese cows.

### Rumen metabolome

**Fig. 6:**
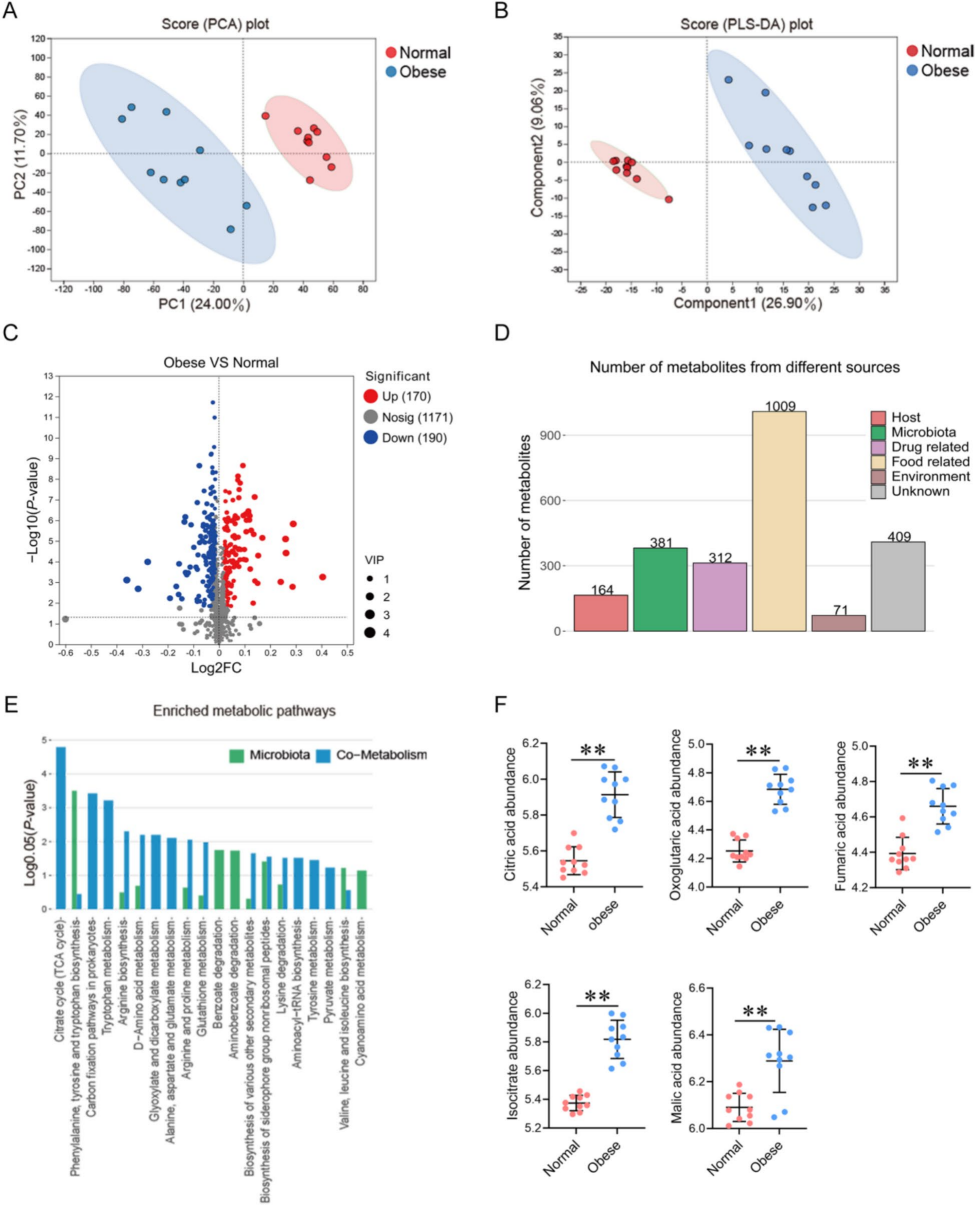
Rumen metabolome of normal and obese dairy cows. **A** The PCA analysis based on metabolites. **B** The PLS-DA analysis based on metabolites. **C** Volcano plot of rumen metabolites. **D** Number of metabolites from different sources. **E** Metabolic pathway enrichment analysis according to different categories of metabolites belonging to the host and microbiota (Only top 20). **F** Significantly different metabolites in the “Citrate cycle (TCA cycle)”. *n* = 10 per group. Significant differences were tested by the Wilcoxon rank-sum test. Data with error bars were expressed as mean ± SD. *P < 0.05, **P < 0.01

Next, we conducted metabolite profiling in rumen fluid samples from normal and obese cows based on untargeted metabolomics. A high Pearson correlation among ruminal QC samples and a tight cluster of QC samples in PCA score plot were observed (Fig. S4A–C), which confirmed the reliability of the data quality. PCA showed that the ruminal samples of obese cows were significantly separated from the samples of normal cows (Fig. 6A; R2X (cum) = 0.459 > 0.4). Moreover, PLS-DA also separated the obese cow samples from normal cow samples (Fig. 6B; R2Y (cum) = 0.999 and Q2 (cum) = 0.94). A total of 1531 metabolites were detected (Table S6), of which 360 exhibited significant differences between the two groups (Fig. 6C; *P* < 0.05 and VIP > 1). In obese cows, 190 metabolites were significantly lower, whereas 170 metabolites were significantly higher than that in normal cows. Furthermore, we identified the sources of the metabolites, finding 164 from the host, 381 from microbiota, 312 from drugs, 1009 from food, 71 from environmental factors, and 409 from unknown sources (Fig. 6D; Table S7). A total of 69 pathways were identified, including 49 pathways from co-metabolism, 17 from microbiota, and 3 from the host (Table S8). Differential metabolites were significantly enriched in the "Citrate cycle (TCA cycle)" (Fig. 6E; *P* < 0.01). We therefore focused on this metabolic pathway and found that 5 metabolites, including citric acid, oxoglutaric acid, fumaric acid, isocitrate, malic acid, were higher in obese cows (Fig. 6F;* P* < 0.01 and VIP > 1).

### Correlation analysis between rumen bacteria, metabolites, and metabolic parameters

**Fig. 7:**
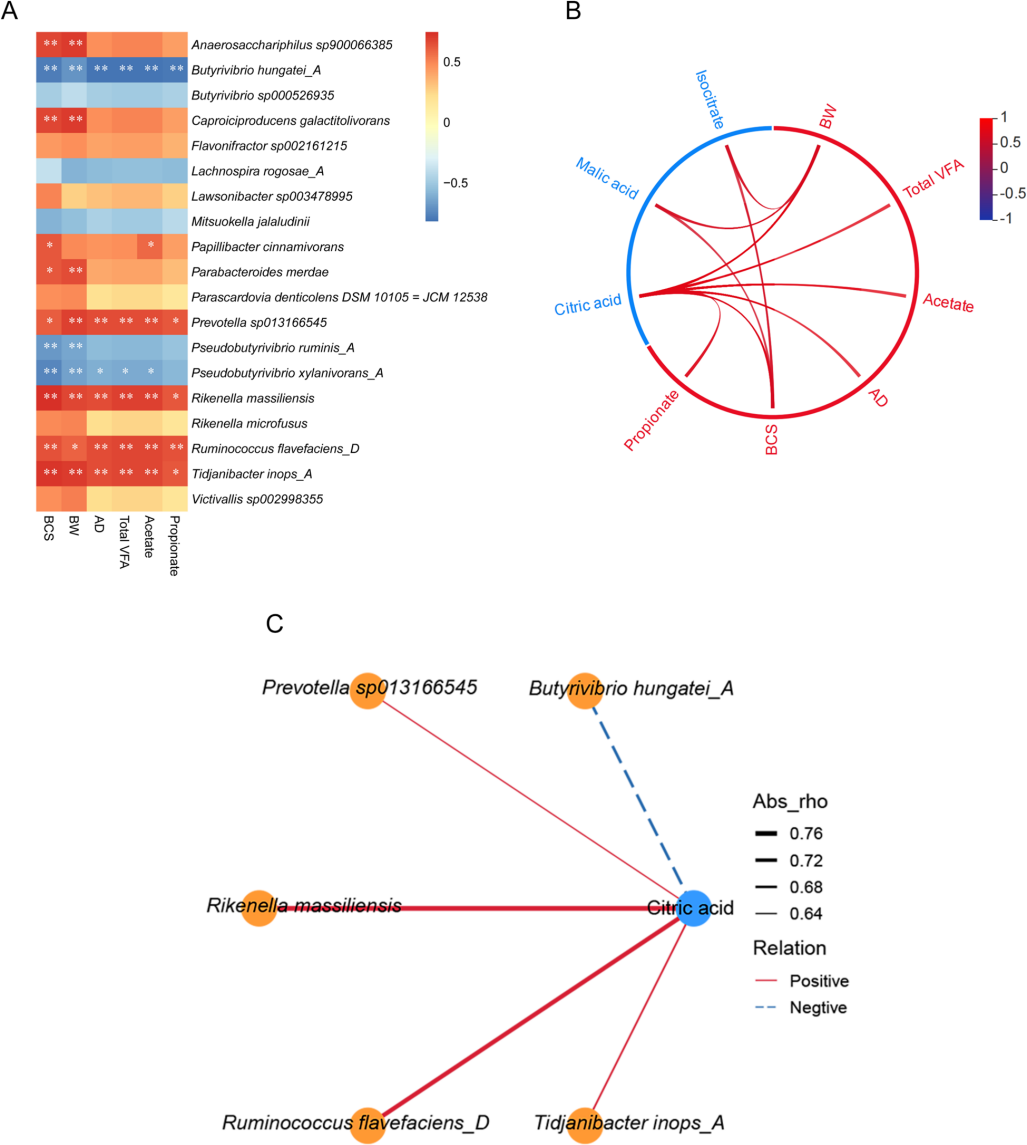
Analysis of Spearman’s correlation between rumen bacterial flora, metabolites and metabolic parameters. **A** Heatmap of correlation between differential bacterial species, phenotype data (BCS, BW, and AD), and differential rumen fermentation parameters. **B** Correlation analysis between differential metabolites (TCA cycle), phenotype data, and differential rumen fermentation parameters. **C** Correlation network showed the association between differential bacteria and metabolites (TCA cycle). The red rectangles or edges represent a positive correlation, while the blue rectangles or edges represent a negative correlation. Correlations with |rho|> 0.6 were presented in (**B**) and (**C**). Correlations with |rho|> 0.6 and *P* < 0.05 were considered significant correlations. *P < 0.05, **P < 0.01

Correlation analysis was conducted between differential bacterial species, phenotype data (BCS, BW, and AD), and differential rumen fermentation parameters (Fig. 7A). The results showed BCS was positively correlated with *Tidjanibacter inops*_*A*, *Ruminococcus flavefaciens*_*D*, *Rikenella massiliensis*, *Prevotella sp013166545*, *Parabacteroides merdae*, *Papillibacter cinnerivorans*, *Caproiciproducens galactitolivorans*, and *Anaerosaccharphilus sp900066385* (|rho*|*> 0.6 and* P* < 0.05 or 0.01), but negatively associated with *Pseudobutyrivibrio ruminis_A*, *Pseudobutyrivibrio xylanivorans_A*, and *Butyrivibrio hungatei_A* (|rho|> 0.6 and *P* < 0.01). BW was positively correlated with *Tidjanibacter inops*_*A*, *Ruminococcus flavefaciens*_*D*, *Rikenella massiliensis*, *Prevotella sp013166545*, *Parabacteroides merdae*, *Caproiciproducens galactitolivorans*, and *Anaerosaccharphilus sp900066385* (*|*rho*|*> 0.6 and* P* < 0.05 or 0.01), but negatively associated with *Pseudobutyrivibrio ruminis*_*A*, *Pseudobutyrivibrio xylanivorans*_*A*, and *Butyrivibrio hungatei*_*A* (|rho|> 0.6 and *P* < 0.01). Total VFA, AD, and acetate were positively correlated with *Tidjanibacter inops*_*A*, *Ruminococcus flavefaciens*_*D*, *Rikenella massiliensis*, and *Prevotella sp013166545* (|rho|> 0.6 and *P* < 0.05 or 0.01), but negatively associated with *Pseudobutyrivibrio xylanivorans*_*A* and *Butyrivibrio hungatei*_*A* (|rho|> 0.6 and *P* < 0.05 or 0.01). Propionate was positively correlated with *Tidjanibacter inops_A*, *Ruminococcus flavefaciens_D*, *Rikenella massiliensis*, and *Prevotella sp013166545* (|rho| > 0.6 and *P* < 0.05 or 0.01), but negatively associated with *Butyrivibrio hungatei_A* (|rho| > 0.6 and *P* < 0.01).

The correlation analysis between differential metabolites (TCA cycle), phenotype data, and differential rumen fermentation parameters is shown in Fig. 7B. Isocitrate and malic acid were positively correlated with BCS and BW (|rho|> 0.6 and *P* < 0.05 or 0.01). Citric acid was positively correlated with BCS, BW, AD, total VFA, acetate, and propionate (|rho|> 0.6 and *P* < 0.01).

Six bacteria (*Tidjanibacter inops*_*A*, *Ruminococcus flavefaciens D*, *Rikenella massiliensis*, *Pseudobutyrivibrio xylanivorans_A*, *Prevotella sp013166545*, and *Butyrivibrio hungatei_A*) and one metabolite (citric acid) showed a close correlation with most phenotype data (Fig. 7A and B). We therefore analyzed the correlation between these six bacteria and the metabolite. Citric acid was negatively associated with *Butyrivibrio hungatei*_*A* (|rho|> 0.6 and *P* < 0.01) but positively associated with *Tidjanibacter inops*_*A*, *Ruminococcus flavefaciens*_*D*, *Rikenella massiliensis*, and *Prevotella sp013166545* (|rho|> 0.6 and* P* < 0.01; Fig. 7C).

## Discussion

**Fig. 8:**
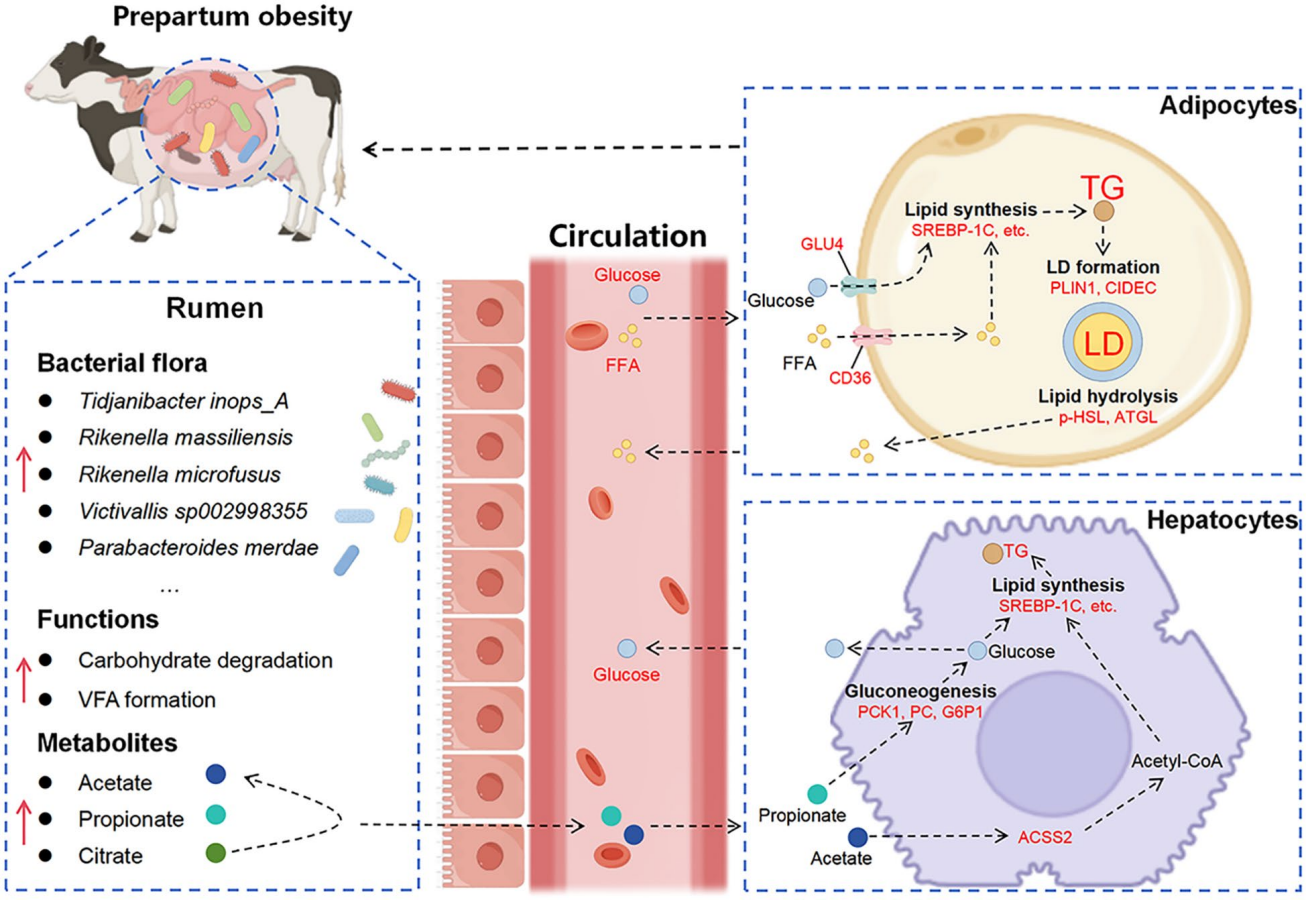
A schematic illustration that depicts the association between rumen bacterial flora, functions and metabolites, and adipocyte lipid deposition in obese dairy cows. The rumen of prepartum obese dairy cows exhibited a higher abundance of VFA-producing bacteria and an increased capacity for carbohydrate degradation and VFA formation. The acetate and propionate produced in the rumen were utilized by the liver for glucose and TG synthesis. Moreover, the elevated levels of circulating glucose and FFA contributed to lipid accumulation in adipocytes, leading to obesity in dairy cows. *VFA* volatile fatty acids, *FFA* free fatty acids, *TG* triglyceride, *LD* lipid droplet, *SREBP*-*1C* sterol regulatory element-binding protein 1C, *ACSS2* acyl-coA synthetase short chain family member 2, *CD36* cluster of differentiation 36, *PCK1* phosphoenolpyruvate carboxykinase 1, *PC* pyruvate carboxylase, *G6P1* glucose-6-phosphatase 1, *GLU4* glucose transporter type, *PLIN1* perilipin 1, *CIDEC* cell death-inducing DNA fragmentation factor-α-like effector c, *p*-*HSL* phosphorylated-hormone-sensitive lipase, *ATGL* adipose triglyceride lipase. Red arrow and font indicate upregulation in prepartum obese cows

In cows, prepartum obesity is emerging as a noteworthy metabolic disorder associated with an increased risk of adverse periparturient outcomes [5, 6]. The rumen microbiota plays a central role in digestion processes, with its fermentation products serving as the primary energy source for the dairy cows [31]. In this study, we observed that obese cows exhibited altered rumen bacterial composition and functions, leading to enhanced acetate and propionate production, which promoted hepatic TG and glucose synthesis. The elevated circulating levels of glucose and FFA contributed to enhanced lipid accumulation in adipocytes of obese cows (Fig. 8). These findings provide potential bacterial regulatory strategies to help manage the prevalence of prepartum obesity in cows.

Alterations in the rumen microbiome are linked to feed efficiency, methane emissions, and lactation performance of dairy cows [32]. *Firmicutes* and *Bacteroidetes*, accounting for > 85% of the rumen microbiota in dairy cows, are pivotal for the degradation of dietary carbohydrates and proteins [33, 34]. In the present study, consistent with previous findings in obese humans [35], the ratio of *Firmicutes* to *Bacteroidetes* was decreased in obese dairy cows. Of note, several members of the *Bacteroidetes*, including *Tidjanibacter inops_A*, *Rikenella massiliensis*, *Papillibacter cinnamivorans*, and *Parabacteroides merdae*, were enriched in obese dairy cows and exhibited positive correlations with both ruminal VFA concentration and BCS. Since the DMI did not differ between normal and obese dairy cows, the observed changes in rumen bacterial flora may contribute to enhanced VFA production and fat deposition in obese dairy cows.

In agreement with study on the gut microbiome of obese mice [36], metagenomic analysis revealed enrichment of pathways related to carbohydrate metabolism and VFA formation in the rumen microbiota of obese dairy cows. Moreover, the enhanced functions of “Starch and sucrose metabolism” and “Glycolysis/gluconeogenesis” suggested that the rumen microbiota of obese dairy cows exhibits an enhanced capacity for carbohydrate degradation. Rumen microbes are equipped with diverse CAZymes for breaking down the complex carbohydrates. Consistent with the results in the obese mice [25], we observed an enrichment of genes encoding CAZymes for carbohydrate deconstruction in the rumen microbiota of obese dairy cows. Furthermore, the abundance of genes involved in VFA production was upregulated in dairy cows with obesity, indicating enhanced microbial fermentation to harvest energy from feed in the form of VFA.

As a result of alternation in microbial composition and function, differences in ruminal metabolomic profiles between normal and obese dairy cows were observed in this study. Apart from VFA, differential metabolites were primarily enriched in the “TCA cycle,” a key pathway in carbohydrate metabolism. A previous study has demonstrated that enrichment of the “TCA cycle” pathway is associated with enhanced digestion rate of carbohydrates in sheep [37]. In line with the upregulation of genes involved in carbohydrates degradation and VFA production, differential metabolites of “TCA cycle” were found to be higher in obese dairy cows, supporting the view of enhanced carbohydrate metabolism in the rumen. Notably, elevated concentration of ruminal citric acid, the initial intermediate of “TCA cycle,” was positively correlated with BCS, BW, AD, ruminal VFA concentration, and the abundance of VFA-producing bacteria. It has been reported that dietary supplementation with citric acid increases ruminal VFA concentration in steers, likely due to enhanced microbial conversion of citric acid to acetate [38]. Consistently, elevated ruminal concentrations of citric acid, acetate and VFA were observed in obese dairy cows. Thus, the ruminal microbiota in obese dairy cows generates elevated levels of “TCA cycle” intermediates, particularly citric acid, resulting in enhanced VFA production.

Previous studies have demonstrated that ruminal VFA can be absorbed across the rumen epithelium and enter systemic circulation [39]. In the liver, propionate is a substrate for gluconeogenesis to produce glucose, and acetate mainly participates in fatty acid synthesis. In bovine hepatocytes, the expression of key gluconeogenic genes *PCK1* and *PC* was induced by propionate in a concentration-dependent manner [40]. Ju et al. [41] reported that dietary supplementation with citrus peel extract enhanced hepatic gluconeogenesis via increasing ruminal propionate production in dairy cows. Consistently, an upregulated hepatic mRNA abundance of *PCK1*, *PC*, and *G6P1*, along with increased concentrations of plasma glucose and ruminal propionate, was observed in obese dairy cows. ATP-citrate lyase (ACLY) is a crucial lipogenic enzyme that links cellular glucose catabolism and de novo lipid synthesis [42]. An upregulated abundance of *ACLY*, coupled with an increased TG content, suggests enhanced glucose-dependent lipogenesis in the liver of obese dairy cows. Acetyl-CoA synthetase ACSS2, which catalyze the conversion of acetate to acetyl-CoA, play a critical role in mediating de novo lipogenesis by providing acetyl-CoA as a key substrate [43]. A study on mice demonstrated that acetate produced by gut microbiota induces the expression of hepatic *ACSS2* [44]. It is likely that the upregulation of *ACSS2* was due to an increased ruminal acetate concentration in obese dairy cows. Moreover, mRNA abundance of *SREBP*-*1C* and its downstream lipogenic targets *FAS* and acetyl coenzyme A carboxylase 1 (*ACC1*) were increased in the liver of obese dairy cows, which is consistent with enhanced hepatic TG content. These findings suggest that increased ruminal acetate and propionate levels in obese dairy cows promote glucose and TG production in the liver.

Previous studies revealed that acetate promotes adipocyte differentiation and lipid deposition in obese mice and rabbit adipose-derived stem cells [45, 46]. However, acetate does not stimulate lipogenesis of adipose tissue in the nonlactating dairy cows [47]. Although elevated ruminal acetate concentration and upregulated mRNA abundance of lipogenic genes of adipose tissue were observed in obese dairy cows, the effect of acetate on bovine adipocyte lipogenesis requires further investigation. Circulating glucose and FFA can be uptake by adipocytes for lipid storage [48]. The present study found an upregulation of mRNA abundance of *GLU4* and *CD36* in the adipose tissue, suggesting that the uptake of glucose and FFA from blood into adipocytes was increased in obese dairy cows. Thus, glucose derived from hepatic gluconeogenesis and FFA serve as critical precursors for lipid accumulation in the adipose tissue of obese dairy cows.

Fatty acids released from lipolysis of adipose tissue are the major source of circulating FFA [49]. The results of this study are well in agreement with studies in mice and humans [50, 51], which also found an elevated plasma FFA level and an increased expression of ATGL and p-HSL, key enzymes involved in intracellular lipolysis. Previous studies demonstrated that increased blood levels of saturated fatty acids are a significant risk factor for obesity [52]. Palmitic acid (C16:0), the most common saturated fatty acid, have been shown to exhibit pronounced lipotoxicity and induce insulin resistance in adipocytes, hepatocytes, and skeletal muscle cells [53, 54]. Consistent with previous studies in humans [55], obese dairy cows exhibited elevated plasma concentrations of palmitic acid and insulin. Thus, it is possible elevated palmitic acid level impaired insulin sensitivity of adipose tissue in obese dairy cows, thereby exacerbating lipid synthesis in adipocytes.

## Conclusions

Our results revealed that VFA-producing bacteria, along with functionality in carbohydrate degradation and VFA formation, were enriched in the rumen microbiota of obese dairy cows, leading to elevated acetate, propionate, and citric acid levels, which contributed to hepatic glucose and TG production. Moreover, increased circulating glucose and FFA were uptake by adipocytes for lipid synthesis, leading to obesity. The results of the current study provided new insights into the ruminal microbial features associated with adipocytes lipid deposition in dairy cows, which may inform future interventions to improve energy metabolism in dairy cows.

## Supplementary Information

Additional file 1. Table S1. Composition and nutrient levels of basal diets. Table S2. Primer sequences are used for real-time PCR analysis. Table S3. The FFA concentration was calculated as the mean value of normal and obese cows. Table S4. Composition of CAZymes based on class-level and family-level enzymes. Table S5. KEGG pathways identified in the metagenomes. Table S6. Number of identified differential metabolites in normal and obese cows. Table S7. Sources of the identified metabolites. Table S8. The enrichment analysis of the metabolites from microbiota, co-metabolism and host.

Additional file 2. Fig. S1. Performance and blood parameters of dairy cows. (A–C) Body condition score (BCS), body weight (BW) and dry matter intake (DMI). (D, E) Activities of aspartate aminotransferase (AST) and alanine aminotransferase (ALT) in the plasma. (F–J) Concentrations of glucose, insulin, triglyceride (TG), free fatty acids (FFA) and beta-hydroxybutyrate (BHBA) in the plasma. *n* = 10 per group. Significant differences were tested using the independent samples *t*-test. Data with error bars were expressed as mean ± SD. ^*^P < 0.05, ^**^P < 0.01. Fig. S2. Differences in the lipid and glucose metabolism of the liver between normal and obese dairy cows. (A) Representative images of hematoxylin–eosin (H&E) and Oil-Red O staining of liver sections. (B) Hepatic TG content. (C) Heatmap of the abundance of genes related to lipid and glucose metabolism in the liver.* n* = 10 per group. Significant differences were tested using the independent samples *t*-test. Data with error bars were expressed as mean ± SD. ^*^P < 0.05, ^**^P < 0.01. Fig. S3. Comparison of functions based on the eggNOG database between normal and obese dairy cows. (A) Statistics of the functional modules. (B) Different functions based on the visualized PCoA. (C) Comparison of the differences in carbohydrate transport and metabolism. *n* = 10 per group. Significant differences were tested by the Wilcoxon rank-sum test. Data with error bars were expressed as mean ± SD. ^*^P < 0.05, ^**^P < 0.01. Fig. S4. Data quality checks. (A) The Pearson correlation of ruminal QC samples. (B, C) The PCA analysis for normal and obese samples containing QC samples. *n* = 10 per group. QC, quality control.

## Data Availability

Raw sequencing data of all 16S rRNA sequences and metagenomes have been deposited into the National Center for Biotechnology Information (NCBI) Sequence Read Archive (SRA) under BioProject accession numbers PRJNA1280256, PRJNA1280522 and PRJNA1280523.

## Abbreviations

AA: Auxiliary activity
ACC1: Acetyl coenzyme A carboxylase 1
ACLY: ATP-citrate lyase
ACOX1: Acyl-CoA oxidase 1
ACSS2: Acyl-coA synthetase short chain family member 2
ACTB: Actin beta
AD: Adipocyte diameter
AKT: Protein kinase B
ALT: Alanine aminotransferase
ACSL1: Long-chain acyl-CoA synthetase 1
AST: Aspartate aminotransferase
ATGL: Adipose triglyceride lipase
BCA: Bicinchoninic acid
BCS: Body condition score
BH: Benjamini-Hochberg
BHBA: Beta-hydroxybutyrate
CAZymes: Carbohydrate active enzymes
CBM: Carbohydrate-binding module
CD36: Cluster of differentiation 36
CE: Carbohydrate esterase
CIDEC: Cell death-inducing DNA fragmentation factor-α-like effector C
CPT1: Carnitine palmitoyltransferase I
CPT2: Carnitine palmitoyltransferase Ⅱ
DMI: Dry matter intake
FAMEs: Fatty acid methyl esters
FAS: Fatty acid synthase
FFA: Free fatty acids
GAPDH: Glyceraldehyde 3-phosphate dehydrogenase
GH: Glycoside hydrolase
GLU4: Glucose transporter type 4
GT: Glycosyltransferase
G6P1: Glucose-6-phosphatase 1
HE: Hematoxylin-eosin
HSL: Hormone-sensitive lipase
LDA: Linear discriminant analysis
NH_3_-N: Ammonia-N
p-AKT: Phosphorylated-AKT
PC: Pyruvate carboxylase
PCA: Principal components analysis
PCK1: Phosphoenolpyruvate carboxykinase 1
PCoA: Principal coordinate analysis
p-HSL: Phosphorylated-HSL
PL: Polysaccharide lyase
PLIN1: Perilipin 1
PLS-DA: Partial least squares discriminant analysis
PPARα: Peroxisome proliferator-activated receptor alpha
SREBP-1C: Sterol regulatory element-binding protein 1C
TG: Triglyceride
TMR: Total mixed ration
VFA: Volatile fatty acid
VIP: Variable importance in the projection
