# Supplementary material for: Altered rumen bacterial flora is associated with increased lipogenesis of adipose tissue in obese dairy cows before calving
Source: Microbiome. 2026 Feb 6;14:66. doi: 10.1186/s40168-026-02343-7 (PMC12895798; doi:10.1186/s40168-026-02343-7)
Supplement: Supplementary file 3 — Additional file 2. Fig. S1. Performance and blood parameters of dairy cows. (A–C) Body condition score (BCS), body weight (BW) and dry matter intake (DMI). (D, E) Activities of aspartate aminotransferase (AST) and alanine aminotransferase (ALT) in the plasma. (F–J) Concentrations of glucose, insulin, triglyceride (TG), free fatty acids (FFA) and beta-hydroxybutyrate (BHBA) in the plasma. n = 10 per group. Significant differences were tested using the independent samples t-test. Data with error bars were expressed as mean ± SD. *P < 0.05, **P < 0.01. Fig. S2. Differences in the lipid and glucose metabolism of the liver between normal and obese dairy cows. (A) Representative images of hematoxylin–eosin (H&E) and Oil-Red O staining of liver sections. (B) Hepatic TG content. (C) Heatmap of the abundance of genes related to lipid and glucose metabolism in the liver. n = 10 per group. Significant differences were tested using the independent samples t-test. Data with error bars were expressed as mean ± SD. *P < 0.05, **P < 0.01. Fig. S3. Comparison of functions based on the eggNOG database between normal and obese dairy cows. (A) Statistics of the functional modules. (B) Different functions based on the visualized PCoA. (C) Comparison of the differences in carbohydrate transport and metabolism. n = 10 per group. Significant differences were tested by the Wilcoxon rank-sum test. Data with error bars were expressed as mean ± SD. *P < 0.05, **P < 0.01. Fig. S4. Data quality checks. (A) The Pearson correlation of ruminal QC samples. (B, C) The PCA analysis for normal and obese samples containing QC samples. n = 10 per group. QC, quality control. [file 40168_2026_2343_MOESM2_ESM.docx]

**Supplementary Figures**


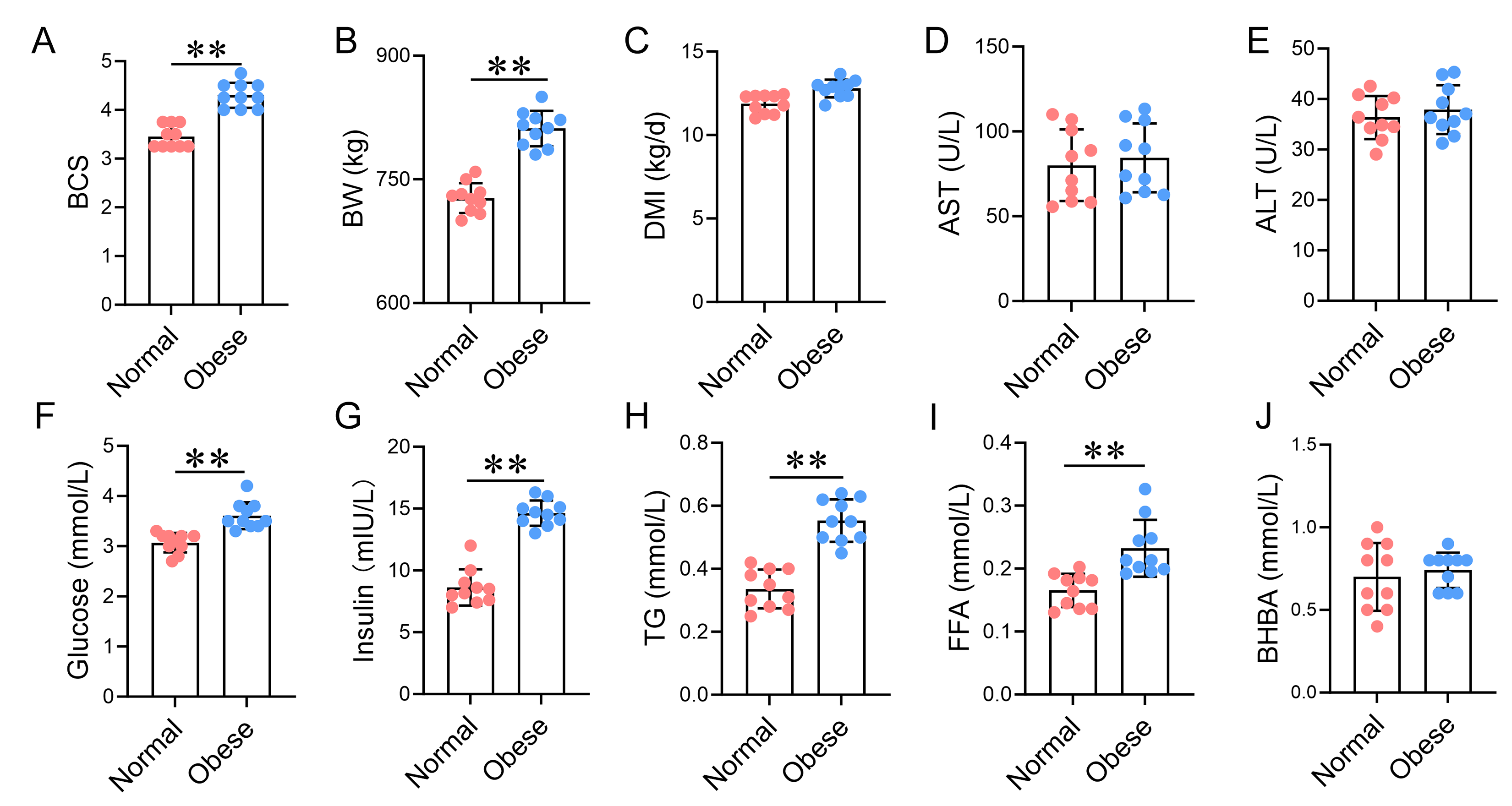


**Fig. S1. Performance and blood parameters of dairy cows. (A**–**C)** Body condition score (BCS), body weight (BW) and dry matter intake (DMI). **(D, E)** Activities of aspartate aminotransferase (AST) and alanine aminotransferase (ALT) in the plasma. **(F**–**J)** Concentrations of glucose, insulin, triglyceride (TG), free fatty acids (FFA) and beta-hydroxybutyrate (BHBA) in the plasma. *n* = 10 per group. Significant differences were tested using the independent samples *t*-test. Data with error bars were expressed as mean ± SD. ^*^*P <* 0.05, ^**^*P <* 0.01.

**
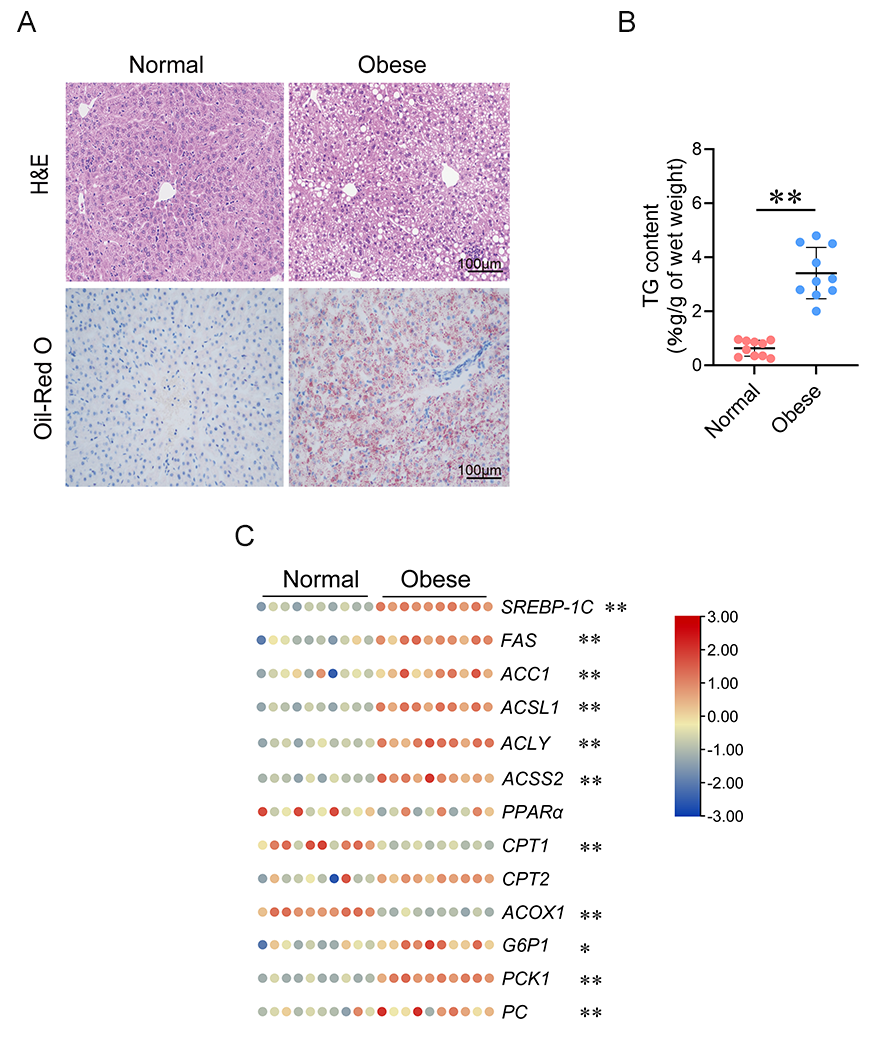
**

**Fig. S2. Differences in the lipid and glucose metabolism of the liver between normal and obese cows. (A)** Representative images of hematoxylin-eosin (H&E) and Oil-Red O staining of liver sections. **(B)** Hepatic TG content. **(C)** Heatmap of the abundance of genes related to lipid and glucose metabolism in the liver**.** *n* = 10 per group. Significant differences were tested using the independent samples *t*-test. Data with error bars were expressed as mean ± SD. ^*^*P <* 0.05, ^**^*P <* 0.01.

**
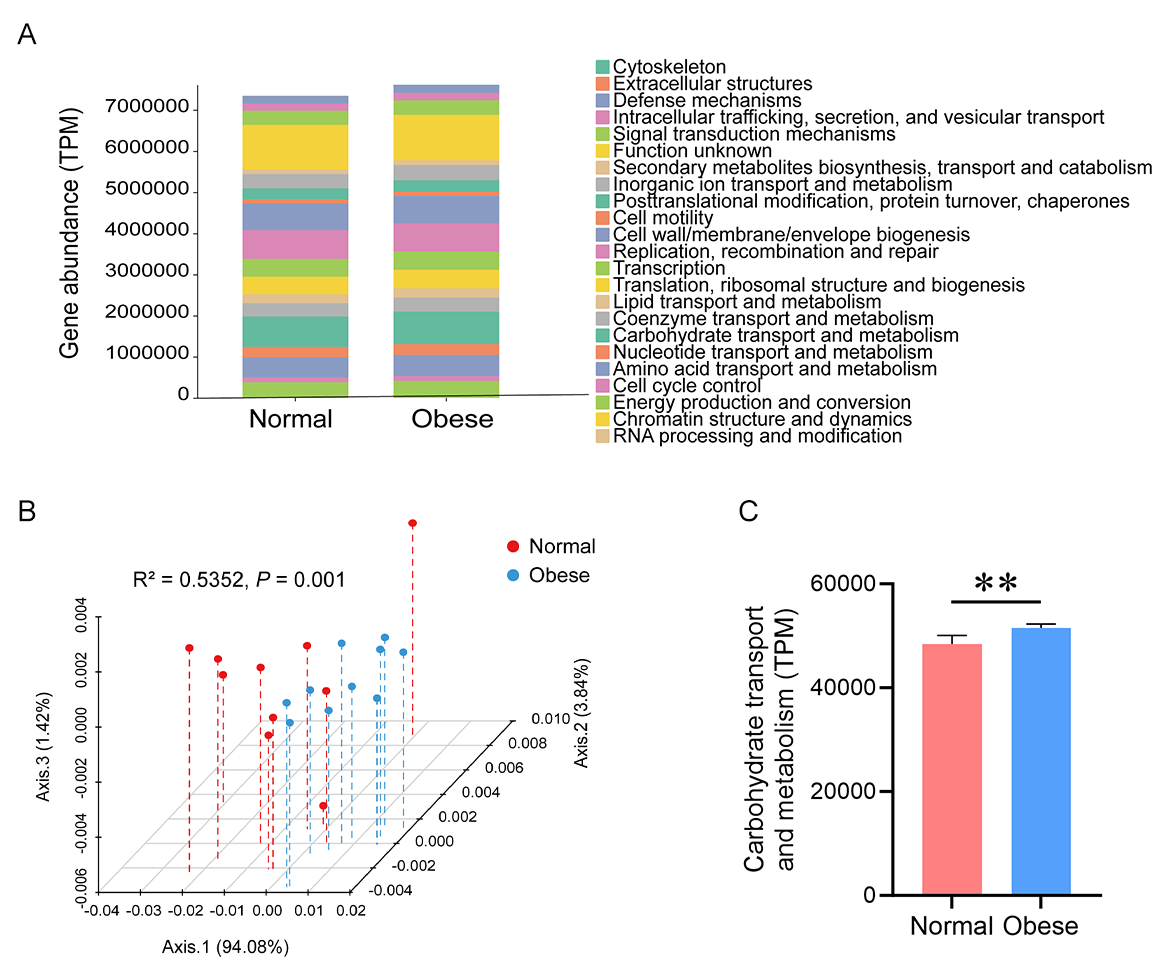
**

**Fig. S3. Comparison of functions based on the eggNOG database between normal and obese cows. (A)** Statistics of the functional modules. **(B)** Different functions based on the visualized PCoA. **(C)** Comparison of the differences in carbohydrate transport and metabolism**.** *n* = 10 per group. Significant differences were tested by the Wilcoxon rank-sum test. Data with error bars were expressed as mean ± SD. ^*^*P <* 0.05, ^**^*P <* 0.01.


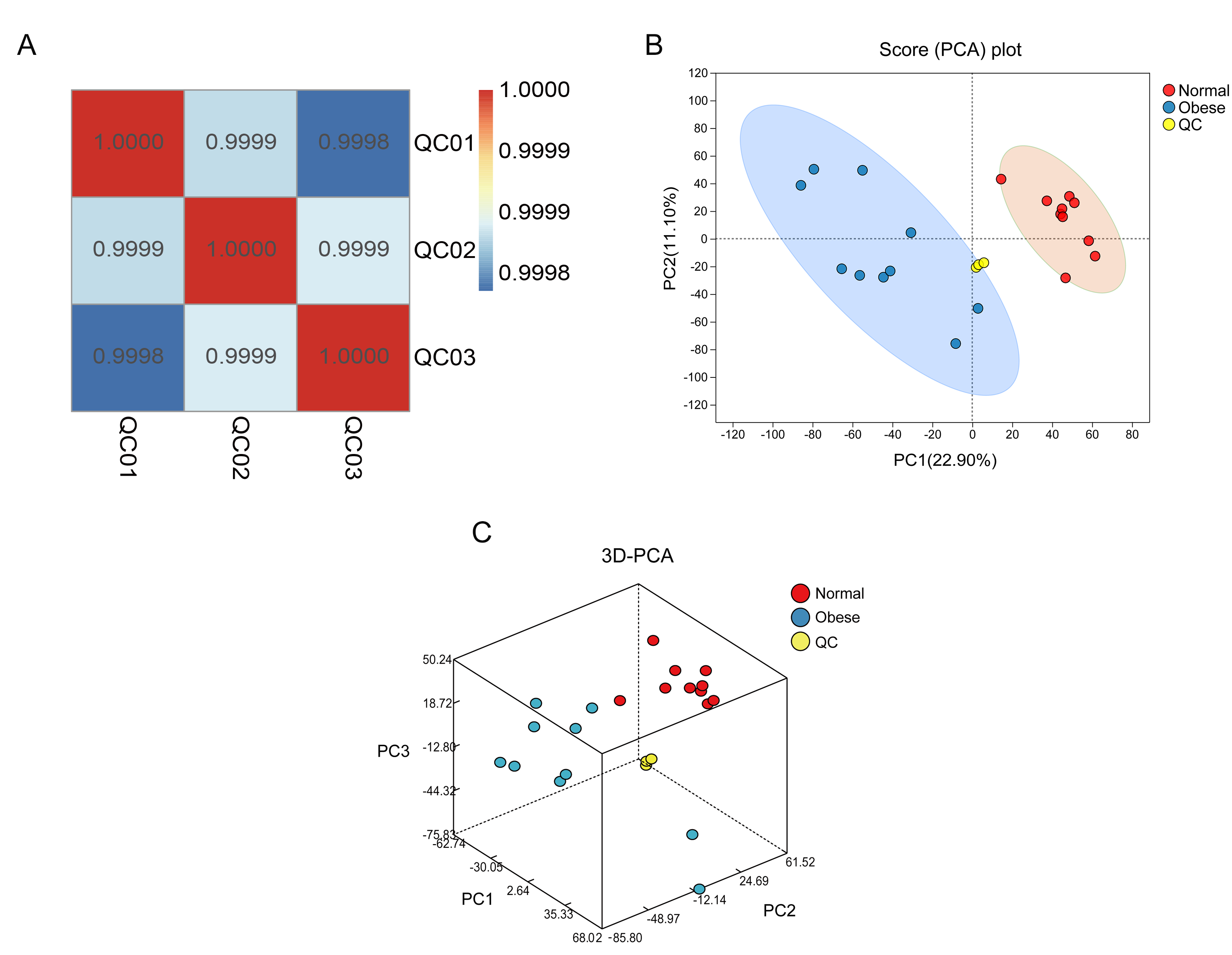


**Fig S4. Data quality checks. (A)** The Pearson correlation of ruminal QC samples. **(B, C)** The PCA analysis for normal and obese samples containing QC samples. *n* = 10 per group. QC, quality control.
